# In silico and multi-spectroscopic analyses on the interaction of 5-amino-8-hydroxyquinoline and bovine serum albumin as a potential anticancer agent

**DOI:** 10.1038/s41598-021-99690-2

**Published:** 2021-10-12

**Authors:** Waralee Ruankham, Kamonrat Phopin, Ratchanok Pingaew, Supaluk Prachayasittikul, Virapong Prachayasittikul, Tanawut Tantimongcolwat

**Affiliations:** 1 Center for Research and Innovation, Faculty of Medical Technology, Mahidol University, Bangkok, 10700 Thailand; 2 Department of Clinical Microbiology and Applied Technology, Faculty of Medical Technology, Mahidol University, Bangkok, 10700 Thailand; 3 Department of Chemistry, Faculty of Science, Srinakharinwirot University, Bangkok, 10110 Thailand; 4 Center for Data Mining and Biomedical Informatics, Faculty of Medical Technology, Mahidol University, Bangkok, 10700 Thailand

**Keywords:** Biophysics, Drug discovery

## Abstract

5-Amino-8-hydroxyquinoline (5A8HQ), an amino derivative of 8-hydroxyquinoline, has become a potential anticancer candidate because of its promising proteasome inhibitory activity to overcome and yet synergize bortezomib for fighting cancers. Therefore, in this study, its physicochemical properties and interaction activities with serum protein have extensively been elucidated by both in vitro and in silico approaches to fulfill the pharmacokinetic and pharmacodynamic gaps. 5A8HQ exhibited the drug-likeness properties, where oral administration seems to be a route of choice owing to its high-water solubility and intestinal absorptivity. Multi-spectroscopic investigations suggested that 5A8HQ tended to associate with bovine serum albumin (BSA), a representative of serum protein, via the ground-state complexation. It apparently bound in a protein cleft between subdomains IIA and IIIA of BSA as suggested by the molecular docking and molecular dynamics simulations. The binding was mainly driven by hydrogen bonding and electrostatic interactions with a moderate binding constant at 10^4^ M^−1^, conforming with the predicted free fraction in serum at 0.484. Therefore, 5A8HQ seems to display a good bioavailability in plasma to reach target sites and exerts its potent pharmacological activity. Likewise, serum albumin is a good candidate to be reservoir and transporter of 5A8HQ in the circulatory system.

## Introduction

**Figure 1:**
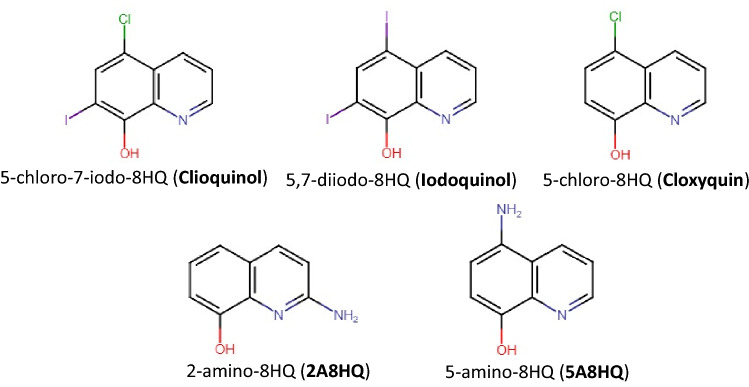
Chemical structures of 8HQ derivatives.

8-Hydroxyquinoline (8HQ) and its derivatives have extensively been advanced for diverse pharmacological activities^1–3^, such as anticancer^4,5^, antibacterial^6–8^, anti-fungal^9^, anti-malarial^10,11^, and anti-neurodegenerative^12^ activities. Presently, some halogenated 8HQs are available as commercial drugs, such as clioquinol (5-chloro-7-iodo-8HQ), cloxyquin (5-chloro-8HQ), and iodoquinol (5,7-diiodo-8HQ)^3^ (Fig. 1). Clioquinol is traditionally used for atopic dermatitis treatment and currently is under decided to reposition as an anti-Alzheimer agent, nonetheless its side effect to cause subacute myelo-optic neuropathy (SMON) is still mysterious. Iodoquinol is effective to treat amoebic infection in the intestine^10^. 5,7-Dichloro-2-[(dimethylamino)methyl]-8HQ or PBT2 has been evaluated under phase II clinical trials to overcome Alzheimer’s and Huntington's diseases^3^. Nitroxoline (5-nitro-8HQ), a nitro derivative, possesses a broad-spectrum antibacterial activity and has been used for treatment and prophylaxis of urinary tract infection for almost 60 years and it has revealed neuroprotective effects in neuronal cells induced by oxidative stress^12^. Presently, amino derivatives of 8HQ have drawn considerable interest, because 2-amino-8HQ (2A8HQ) and 5-amino-8HQ (5A8HQ) exhibit potent antiproliferative activity against a panel of human tumor cells. 5A8HQ can induce cytotoxicity of leukemia and myeloma via a proteasome inhibition mechanism^13^, which can also be observed in lung and ovarian cancer cell lines of human origin^5^. Interestingly, the anti-proteasome activity of 5A8HQ can overcome the resistance to bortezomib, the FDA approved proteasome inhibitor, in some cancer cell lines. 5A8HQ also represents a potent antioxidant activity with an IC_50_ of 8.71 µM, which is better than that of α-tocopherol (13.47 µM)^14^. These promising bioactivities make 5A8HQ highly possible for utilizing as an anticancer agent in the future, where its synergistic effect with bortezomib has been recognized and already patented^15^. Therefore, the basic knowledge about interaction of 5A8HQ with biomolecules is of the utmost importance since it significantly influences efficacy and toxicity of the compound, which is a main cause of clinical trial failures in several promising bioactive agents. Serum albumin^16^, transferrin^17^, and DNA^18^ are often chosen as a primer to explore drug-biomolecule interaction. However, serum proteins are one of the major factors that control the pharmacokinetics and pharmacodynamics, and thereby determine efficiency and safety of the drug. Once entering the blood circulation, some fractions of drug are freely distributed to reach their target sites and exert pharmacological activities as well as toxicities, while some are reversibly bound with serum proteins at the equilibrium condition as inactive forms. Serum proteins generally serve as a reservoir of drugs because drug-bound proteins are tolerant to many elimination systems, e.g., enzymatic hydrolysis in liver and glomerular filtration in kidney that help prolong half-life and maintain activities of drugs^19^.

Albumin is the most abundant protein in serum of mammals, which can be found at a concentration range of 35–50 mg/mL in healthy person to serve as vital physiological functions, such as maintaining homeostasis and oncotic pressure, transporting numerous intrinsic and extrinsic molecules, providing antioxidative and anticoagulant effects, modulating neutrophil adhesion in the inflammatory process, as well as controlling pharmacokinetics and pharmacodynamics of drugs^20,21^. Practically, bovine serum albumin (BSA) is frequently used as a representative of human serum albumin (HSA) in vast varieties of investigations, especially biomedical device development^22^, protein–ligand interaction^23^, and drug delivery studies^24^. BSA is not only similar in composition and structure (~ 76% sequence homology)^25^, but also comparable in physicochemical properties with HSA. It is also easier to access and cheaper than HSA. Albumin is structurally conserved in all mammals in a heart-shaped structure, which consists of three homologous domains (I, II, and III). For BSA, domains I, II, and III are located at residues 1–195, 196–383, and 384–583, respectively. Each domain can be differentiated into subdomains A and B interconnected with the long loop structure^26,27^. Two principal ligand-binding sites are found on albumin structure at the subdomains IIA and IIIA, namely Sudlow’s site I and site II, respectively. Bulky heterocyclic anions preferentially bind to site I with delocalized charges, while site II mainly binds with aromatic carboxylates via a combination of hydrophobic, hydrogen bonding, and electrostatic interactions^28,29^. Besides, long-chain fatty acids are known to be a major ligand of serum albumin, in which seven fatty acid-binding pockets (FAs) are identified at multiple locations of serum albumin denoted as FA1 to FA7^29–31^. Sudlow’s site I is equivalent to FA7 and site II belongs to FA3 and FA4. FAs do not only bind fatty acids, but also other intrinsic and extrinsic molecules, for examples hemin^32^, steroid hormones^33^, and exogenous drugs^34^. Generally, binding of drug with serum albumin is a reversible process, where drug can be released and rebind with serum albumin at an equilibrium, determined by their distinct binding or dissociation constant. The high binding constant value infers that drug strongly binds to serum albumin and high dose of drug is generally required to maintain free fraction and reach therapeutic level for effective treatment. The low binding constant is vice versa, wherein drug weakly binds to serum albumin and is high prevalence in unbound form to exert activity and be eliminated. Thus, understandings of interaction mechanism and binding affinity of drug with serum albumin are important to determine pharmacokinetics and pharmacodynamics that significantly affect on drug efficacy. The molecular and structural insights of drug-protein interaction are also valuable for design and improvement of potential drugs. Therefore, drug-likeness and mechanistic interaction of 5A8HQ with BSA have been extensively explored in this study using both in vitro and in silico approaches. Multi-spectroscopic methods (e.g., absorption, circular dichroism, and fluorescence spectrometry) together with various biophysical models (e.g., Stern–Volmer’s, Hill’s, and thermodynamic models) have been employed to determine interaction mechanism and conformational alteration of BSA and 5A8HQ. Also, their binding pocket and structural stability have been explored by molecular docking and coarse-grained protein modeling tools.

## Results and discussion

### Prediction of physicochemical and pharmacokinetic properties

To evaluate drug-likeness of the 5A8HQ, physicochemical and pharmacokinetic properties were predicted using SwissADME (http://www.swissadme.ch)^35^ and pkCSM (http://biosig.unimelb.edu.au/pkcsm)^36^ servers. 5A8HQ showed feasibility to be a drug-like molecule because its physicochemical properties (Table 1) could attain several drug-likeness filtering criteria, i.e., Lipinski^37^, Ghose^38^, Veber^39^, and Egan^40^ rules. Furthermore, the pharmacokinetic properties of 5A8HQ could be estimated from its predicted ADME (absorption, distribution, metabolism, and excretion) properties, suggesting that 5A8HQ was very soluble in water and could be absorbed by human intestine up to 92% and skin with permeation rate of ‒7.36 cm/s. Also, it could cross the blood–brain barrier; thus, these properties make 5A8HQ suitable to be advanced as an oral drug and possibly effective in the central nervous system (CNS), but further investigation is still required. Also, the predictions showed that 5A8HQ might not be metabolized by the major cytochrome isoenzymes (CYPs). The estimated maximum toleration of 5A8HQ in human was 0.847 log mg/kg/day with a total clearance rate at 0.465 log mL/min/kg. It also revealed no hepatocellular toxicity and no skin sensitization. The unbound fraction of 5A8HQ in human was predicted to be 0.484, suggesting that 5A8HQ was moderately associated with serum proteins, and well available in plasma to exert pharmacological activities on the targets.

**Table 1:** Physicochemical properties of 5A8HQ acquired from SwissADME. MLogP and WLogP are the calculated n-octanol/water partition coefficients derived from Moriguchi’s and Daina’s calculation methods, respectively^35^.

Physicochemical parameters	Value
Molecular weight (g/mol)	160.17
WLogP	1.53
MLogP	0.57
Number of rotatable bonds	0
Number of H-bond acceptors	2
Number of H-bond donors	2
Topological polar surface area (Å^2^)	59.14
Molar refractivity	48.17

### Fluorescence quenching of BSA by 5A8HQ

**Figure 2:**
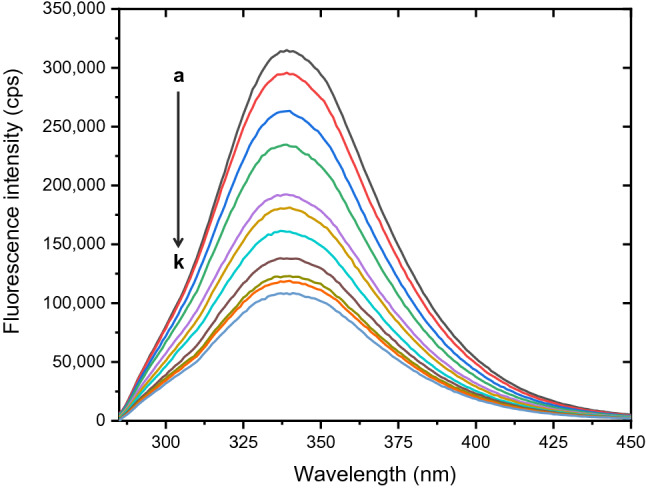
Fluorescence spectra of BSA (4 µM) in equilibration with 5A8HQ (**a**–**k**: 0, 5, 10, 30, 50, 70, 90, 110, 130, 150, and 170 µM) upon excitation with 280 nm ray length at 310 K.

To explore more information on pharmacokinetic behaviors of 5A8HQ, its interaction mechanism with serum proteins was extensively studied using BSA as a model serum protein. Careful analysis of intrinsic fluorescence alterations helps elucidate microenvironmental changes and structural perturbations of a protein structure, which can be caused by several factors, such as subunit association, complex formation, and denaturation^41^. In this study, BSA showed a distinctive fluorescence spectrum with a maximum emission peak at 340 nm (Fig. 2), in which its intensity was apparently diminished when confronted with 5A8HQ as a concentration-dependent manner. Neither peak shift nor spectral shape change was spotted. These findings indicate the interaction phenomenon between BSA and 5A8HQ, which has been later uncovered its mechanistic process by implementing Stern–Volmer analysis on the fluorescence quenching dataset.

Quenching of intrinsic protein fluorescence can be triggered by either static or dynamic process. In some circumstances, both processes can concurrently provoke fluorescence quenching known as a combined quenching mechanism. Static and dynamic quenching processes obey a linear model of the Stern–Volmer equation (Eq. 1), which can be approximately differentiated by scrutinizing the Stern–Volmer constant (${K}_{sv}$) at different temperatures. Positive relationship of ${K}_{sv}$ with temperature indicates dynamic quenching process, while negative association infers static process. Dynamic quenching occurs via collisional encountering among protein and ligand. Increasing temperature enlarges ligand motion to hit protein and quench its fluorescence at the excited state, resulting in increasing ${K}_{sv}$. Static quenching is contrary to the dynamic one. It is arisen by the complexation of protein and ligand, in which rising temperature tends to disfavor binding event and lessen ${K}_{sv}$ value. Combined quenching process influences both ground-state and excited state of protein fluorescence, which usually deviates the linearity of classical Stern–Volmer plot to downward or upward curvature^42^.

1 $$\frac{{F}_{0}}{F}=1+{K}_{sv}\left[Q\right]=1+{k}_{q}{\tau }_{0}\left[Q\right]$$

${F}_{0}$ and $F$ are the steady-state fluorescence intensities of fluorophore (BSA) in the absence and the presence of quencher (5A8HQ), respectively. $\left[Q\right]$ refers to the molar concentration of quencher. ${K}_{sv}$ is Stern–Volmer constant. ${k}_{q}$ and ${\tau }_{0}$ are biomolecular quenching rate constant and fluorescence lifetime of the fluorophore without quencher, respectively.

**Figure 3:**
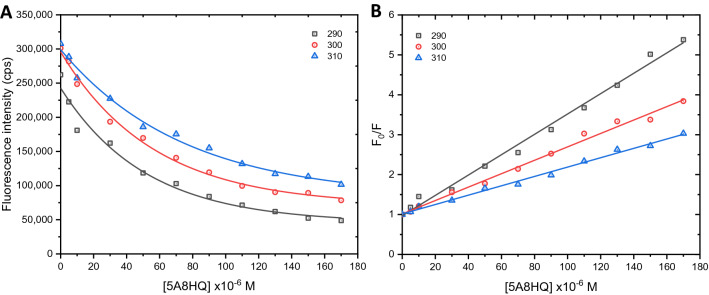
(**A**) Fluorescence quenching and (**B**) Stern–Volmer plots of BSA in the presence of 5A8HQ at 290, 300, and 310 K. [BSA] = 4 µM; [5A8HQ] = 0–170 µM; λ_ex_ = 280 nm; λ_em_ = 340 nm.

Fluorescence quenching and thermodynamic analyses were performed at three different temperatures including 310, 300, and 290 K to imitate physiological, ambient, and hypothermic conditions, respectively. Fluorescence quenching and Stern–Volmer plots of BSA in the presence of 5A8HQ were shown in Fig. 3. The 5A8HQ quenched fluorescence emission of BSA in a concentration dependent manner and almost reached saturation at 5A8HQ concentration above 130 µM (Fig. 3A). The Stern–Volmer plots behaved in a positive linear trend, indicating a single process of BSA fluorescence quenching by 5A8HQ (Fig. 3B). The ${K}_{sv}$ values derived from the plots’ slopes were decreased with increasing temperature of interaction (Table 2), suggesting static quenching process mainly involved in BSA‒5A8HQ interaction.

**Table 2:** Stern–Volmer and quenching rate constants for BSA‒5A8HQ interaction.

T (K)	${K}_{sv} \times$10^4^ (M^−1^)	${k}_{q} \times$10^12^ (M^−1^ s^−1^)	r^2^
290	2.773 ± 0.071	2.773 ± 0.071	0.9935
300	1.303 ± 0.043	1.303 ± 0.043	0.9889
310	1.173 ± 0.032	1.173 ± 0.032	0.9928

In addition, quenching process could be roughly verified by determining the quenching rate constant of the interaction (Eq. (1)), in which the quenching rate constant at 2 × 10^10^ M^−1^ s^−1^ is usually employed as a maximum cutoff for diffusion collision quenching of various quenchers^43^. The fluorescence lifetime of excited state can be measured using a lifetime spectrofluorometer. However, in case of inaccessible to such instrument like this study, a lifetime of 1 × 10^−8^ s is generally assumed for the calculation of quenching rate constant^44^. Accordingly, the quenching rate constant of BSA‒5A8HQ interaction at each temperature was approximated to be 10^12^ M^−1^ s^−1^ (Table 2), which was 100 times higher than that of the typical collisional quenching process (2 × 10^10^ M^−1^ s^−1^). Therefore, this finding indicates that 5A8HQ primarily interacts with BSA by static process through ground-state complex formation. Thereby, the binding constants of complexation were elucidated using Hill’s plot.

### Binding and thermodynamic parameters of BSA‒5A8HQ complex

**Figure 4:**
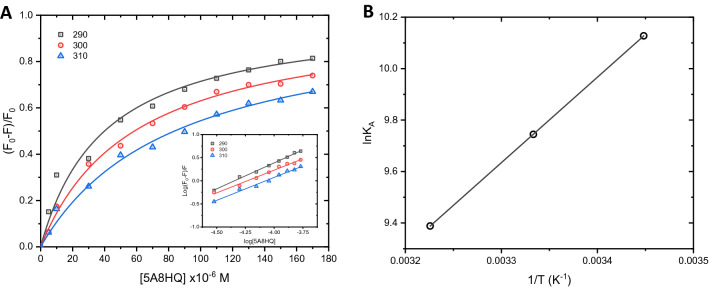
(**A**) Nonlinear Hill’s equation fitted on fluorescence dataset of BSA (4 µM) in the presence of 5A8HQ (0–170 µM) at 290, 300, and 310 K. Inset is a linearized Hill’s plot. (**B**) Van’t Hoff plot of $ln{K}_{A}$ versus $1/T$ for the binding of 5A8HQ with BSA.

Various non-covalent interactions play an important role in the binding of ligand to protein including hydrogen bonding, van der Waals force, electrostatic, and hydrophobic interactions. Therefore, complex formation between BSA and 5A8HQ was further explored to determine principal interaction forces and binding affinity. Nonlinear Hill’s equation (Eq. (2)) was applied on the fluorescence dataset of BSA–5A8HQ complex to obtain binding constant (${K}_{A}$) and Hill coefficient constant ($n$), which fundamentally infer affinity and cooperativity of the binding, respectively (Fig. 4A).

2 $$\frac{{F}_{0}-F}{{F}_{0}}=\frac{{K}_{A}{ [Q]}^{n}}{1+{K}_{A}{[Q]}^{n}}$$

${F}_{0}$, $F$, and $\left[Q\right]$ stand for the same annotations as the aforementioned in Eq. (1). Hill’s equation can be transformed to linear form as an expression of Eq. (3), which is often applied in many studies.

3 $$log\frac{\left({F}_{0}-F\right)}{F}=log{K}_{A}+nlog\left[Q\right]$$

${K}_{A}$ is similar to ${K}_{SV}$ in terms of binding constant, but in different aspects of interaction behavior. ${K}_{A}$ implies a degree of affinity at static process, while ${K}_{SV}$ rather reflects dynamic behavior among protein and ligand^16^. As shown in Table 2 and 3, ${K}_{A}$ and ${K}_{SV}$ values at each temperature of BSA‒5A8HQ equilibrium were nearly similar, suggesting one mechanism majorly involved in such interaction that should be static process or complex formation. The binding constants of BSA‒5A8HQ interaction were found in a moderate level about 10^4^ M^−1^ (Table 3). Increasing temperature resulted in the reduction of binding constant, indicating the thermal destabilization of BSA–5A8HQ complex. Its major interaction force seems to be hydrogen bonding and/or electrostatic interaction because it could significantly be weakened by the rising temperature. In contrast, if the interaction was mainly driven by hydrophobic interaction, enhancement of the binding constant would rather be observed^45^. Hill constants were equal to 1 in all three temperature conditions, indicating that 5A8HQ binds to BSA in a noncooperative manner.

**Table 3:** Binding parameters including binding constant (${K}_{A}$), Hill coefficient constant ($n$), and the relative thermodynamic parameters (Gibbs free energy; $\Delta G^\circ$, enthalpy; $\Delta H^\circ$, and entropy; $\Delta S^\circ$) for the interaction of BSA and 5A8HQ at 290, 300, and 310 K. r^2^ is the squared correlation coefficient.

T (K)	${K}_{A} \times$10^4^ (M^−1^)	$n$	r^2^	$\Delta H^\circ$(kJ mol^−1^)	$\Delta S^\circ$(J mol^−1^ K^−1^)	$\Delta G^\circ$, (kJ mol^−1^)
290	2.502 ± 0.169	1 ± 0	0.9772			‒24.417
300	1.706 ± 0.043	1 ± 0	0.9962	‒27.617	‒11.033	‒24.307
310	1.195 ± 0.001	1 ± 0	0.9910			‒24.197

Thermodynamic parameters are useful to explain the nature of binding force between protein and ligand. Enthalpy ($\Delta H^\circ$) and entropy ($\Delta S^\circ$) of the BSA–5A8HQ interaction were calculated by fitting a Van’t Hoff plot (Fig. 4B) with Eq. (4) and then derived Gibbs free energy ($\Delta G^\circ$) with Eq. (5).

4 $$\mathrm{ln}\,\,{K}_{A}=-\frac{\Delta H}{RT}+\frac{\Delta S}{R}$$

5 $$\Delta G^\circ =\Delta H^\circ -T\Delta S^\circ $$

In Eqs. (4) and (5), ${K}_{A}$ is a binding constant at $T$ temperature (K). $R$ is a gas constant equal to 8.314 J mol^−1^ K^−1^.

The $\Delta G^\circ$ implies the spontaneity of interaction, in which negative and positive values mean spontaneous and nonspontaneous outcomes, respectively. $\Delta H^\circ$ and $\Delta S^\circ$ help determine interaction force of the binding event^46^. Negative $\Delta H^\circ$ and $\Delta S^\circ$ infer the involvement of hydrogen bonding and van der Waals forces. Negative $\Delta H^\circ$ and positive $\Delta S^\circ$ point to electrostatic and ionic interactions. Positive $\Delta H^\circ$ and $\Delta S^\circ$ imply hydrophobic interaction, while negative $\Delta H^\circ$ and positive $\Delta S^\circ$ cannot occur. Herein, interaction of BSA with 5A8HQ showed negative values of $\Delta G^\circ$, $\Delta H^\circ$, and $\Delta S^\circ$, indicating that 5A8HQ spontaneously binds with BSA via mainly hydrogen bonding and van der Waals interactions (Table 3). The complexation was exothermic and enthalpy-driven with Gibbs free energy of about ‒24 kJ mol^−1^.

### Microenvironmental changes nearby tyrosine and tryptophan residues

**Figure 5:**
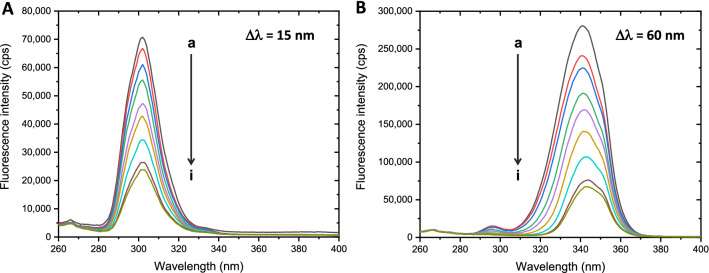
Synchronous fluorescence spectra of BSA (4 µM) when exposed to 5A8HQ (a–i: 0, 5, 10, 30, 50, 70, 110, 150, and 170 µM) at 310 K and 280 nm excitation wavelength. (**A**) Tyrosine spectra (Δλ = 15 nm) and (**B**) tryptophan spectra (Δλ = 60 nm).

Trp and Tyr fluorescence are highly sensitive to microenvironmental changes because of ligand interaction. In many circumstances, binding of the ligand can perturb BSA structure and expose Trp and Tyr with the external milieu. BSA contains 2 Trp residues located interiorly at subdomain IIA (Trp213) and superficially at subdomain IB (Trp134), while its 18 residues of Tyr are found to be distributed along the entire polypeptide chain^47^. Characteristic fluorescence of Tyr and Trp can be observed by synchronous fluorescence analysis, where excitation and emission wavelengths are simultaneously adjusted at the constant offsets (Δλ) of 15 and 60 nm to display Tyr and Trp fluorescence spectra, respectively. Intensity change and peak shift of the spectra infer microenvironmental changes nearby Trp and Tyr residues, where a red-shift suggests the increased polarity, while a blue-shift refers to the increased hydrophobicity. In the absence of 5A8HQ, typical fluorescence characteristics of Tyr and Trp were observed with the maximum emission peaks at 302 and 340 nm, respectively (Fig. 5). 5A8HQ dramatically quenched fluorescence emission of both Tyr and Trp in a concentration dependent manner. A peak shift was not observed in Tyr fluorescence, while a slight red shift from 340 to 344 nm was found in Trp spectra. These findings indicated that 5A8HQ interacts with and perturbs BSA structure, resulting in Trp exposure to polarity or aqueous environment.

### Conformational change analysis on 3D fluorescence spectrum

**Figure 6:**
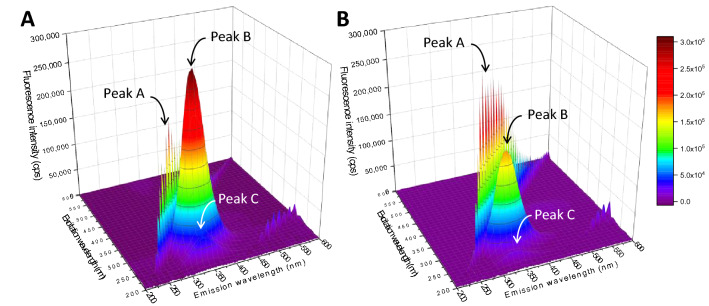
3D fluorescence spectra of BSA (4 µM) in the (**A**) absence and (**B**) presence of 5A8HQ (5 µM) at 310 K.

Three-dimensional (3D) fluorescence spectrum analysis is widely applied to elucidate conformational alteration of protein. As shown in Fig. 6A, BSA exhibited three characteristic peaks on the 3D fluorescence spectrum, including peaks A, B, and C. Peak A is the first-order Rayleigh scattering that appears when excitation (λ_ex_) and emission (λ_em_) wavelengths are equal. Peak B majorly belongs to the Trp and Tyr characteristics, where λ_ex_ is 280 nm and λ_em_ is 340 nm. Peak C is a polypeptide characteristic located at around 236 nm and 335 nm of λ_ex_ and λ_em_, respectively. Intensity change and/or peak shift imply the structural alteration of protein^48^. In the presence of 5A8HQ (Fig. 6B), intensity of peak A was slightly increased, implying that 5A8HQ induces hydrodynamic diameter enlargement of the BSA that in turn enhances the Rayleigh scattering effect^49–51^ as previously observed on other 8HQ derivatives^50^. In addition, 5A8HQ apparently induced intensity losses of peak B and peak C, suggesting that it exerts direct effects on BSA conformation by inducing polypeptide chain alterations and microenvironmental changes around the Trp and Tyr residues.

### Fluorescence resonance energy transfer (FRET) between BSA and 5A8HQ

Besides microenvironmental changes around its fluorophore, quenching of BSA fluorescence may also be provoked by fluorescence resonance energy transfer (FRET) process, in which the excited Trp and/or Tyr transfer their energy to the quencher (5A8HQ) via dipole–dipole interaction. However, the FRET phenomenon is strictly dependent on the degree of overlapping between emission spectrum of fluorophore and absorption spectrum of quencher. Also, fluorophore and quencher must be in proximity (2–8 nm) to each other and their transition dipoles must align in parallel orientation. FRET is a useful method to study molecular distance among molecules that can provide guidance on whether ligand binds with protein at proximity to the Trp fluorophore. The distance ($r$) between quencher and fluorophore can be calculated by Eq. (6).

6 $$E=1-\frac{F}{{F}_{0}}=\frac{{R}_{0}^{6}}{{R}_{0}^{6}-{r}^{6}}$$

$E$ stands for the efficiency of fluorescence resonance energy transfer. ${F}_{0}$ and $F$ are fluorescence intensities of BSA in the absence and presence of 5A8HQ, respectively. ${R}_{0}$ is the Förster distance, where half of the excitation energy is transferred to the acceptor. ${R}_{0}$ can be obtained by Eqs. (7) and (8) as follows:

7 $${R}_{0}=0.211{\left[\frac{{k}^{2}{\varnothing }_{D}J}{{n}^{4}}\right]}^\frac{1}{6}$$

8 $$J=\frac{{\int }_{0}^{\infty }F\left(\lambda \right)\varepsilon \left(\lambda \right){\lambda }^{4}d\lambda }{{\int }_{0}^{\infty }F\left(\lambda \right)d\lambda }$$

where ${k}^{2}$ is the orientation factor of the dipoles, which can range between 0 and 4. ${\varnothing }_{D}$ and $n$ denote the fluorescence quantum yield of donor without quencher and the refractive index of the medium, respectively. $J$ is an overlapping between the donor fluorescence spectrum and the quencher absorption spectrum. $F\left(\lambda \right)$ and $\varepsilon \left(\lambda \right)$ mean fluorescence intensity of the donor and molar absorption coefficient of the acceptor at the $\lambda$ wavelength.

**Figure 7:**
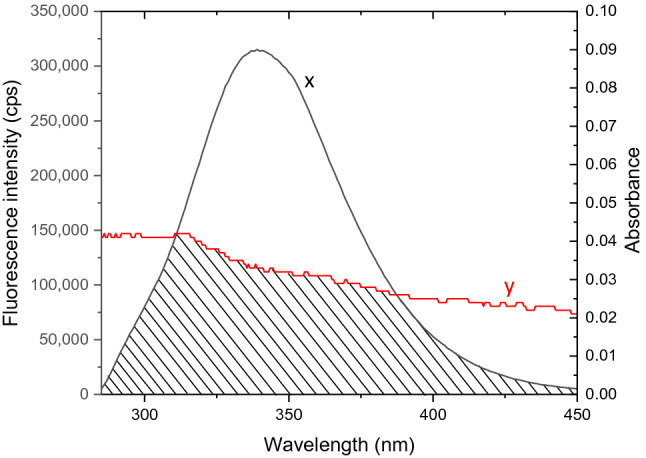
Fluorescence spectrum of BSA (4 µM; x) superimposed with an absorption spectrum of 5A8HQ (5 µM; y). Shade indicates the overlapping area among spectra.

An overlay of the BSA fluorescence and 5A8HQ absorption spectra was shown in Fig. 7. Its integral spectral overlap ($J$) was found to be 9.55 × 10^13^ M^−1^ cm^−1^ nm^4^. ${R}_{0}$ was determined on the assumption that ${\varnothing }_{D}$ and $n$ were 0.15 and 1.336, respectively. However, Förster distance depends greatly on orientation between donor’s and acceptor’s dipoles. ${k}^{2}$ value of 0.667 is generally assumed for dynamic dipole orientation, where dipoles are randomly oriented because of their rotational diffusion. In case of donor‒acceptor orientation is static that does not change over time, ${k}^{2}$ of 0.475 is typically assigned for calculation. Based on these two assumptions of dipole orientation, ${R}_{0}$ were calculated to be 2.37 and 2.50 nm, which subsequently yielded the Trp to 5A8HQ distances ($r$) at 3.01 and 3.18 nm for static and dynamic dipole orientations, respectively. Therefore, the estimated $r$ values of both static and dynamic dipole orientations were in the range of 2–8 nm and in between the 0.5 ${R}_{0}$ and 1.5 ${R}_{0}$ distances, suggesting that 5A8HQ binds on the BSA structure at the site nearby the Trp residue and can acquire excited state energy of Trp via radiationless mechanism.

### Structural stability of BSA against 5A8HQ exposure

**Figure 8:**
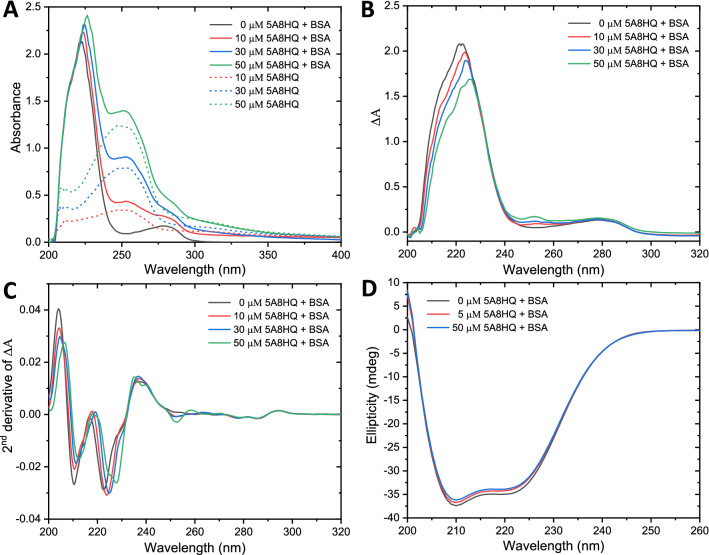
Unpolarized absorption spectra (**A**‒**C**) and circular dichroism spectra (**D**) of BSA (4 µM) in the presence of various 5A8HQ concentrations. (**A**) Original absorption spectra of BSA in equilibration with 5A8HQ (0, 30, and 50 µM) and free 5A8HQ without BSA. (**B**) 5A8HQ-subtracted absorption spectra of BSA‒5A8HQ complex. (**C**) Second-order derivative of the 5A8HQ-subtracted spectra. (**D**) CD spectra of BSA equilibrated with 5A8HQ.

To gain insight into the interaction mechanism between BSA and 5A8HQ, their absorption characteristics toward unpolarized and circularly polarized light were investigated by UV–Vis and circular dichroism spectroscopies. Binding of the ligand can provoke conformational changes and may alter light absorption properties of the host protein. Proteins commonly absorb light at 280 nm, belonging to the characteristic of aromatic side chains of Tyr, Trp, and Phe^52^. Likewise, polypeptide backbone also strongly absorbs UV in a range between 180‒230 nm because of the n‒π* and π‒π* transitions. Herein, BSA exhibited a typical UV absorption characteristic with major and minor peaks at 222 and 278 nm, corresponding to the electronic transitions in peptide bonds and aromatic amino acids, respectively (Fig. 8A,B). In addition, 5A8HQ itself exhibited a major absorption peak at 254 nm. The BSA absorption spectral change was noted after adding 5A8HQ, but it was also overlaid with characteristic band of 5A8HQ (Fig. 8A). Therefore, 5A8HQ absorbance was subtracted out of the BSA‒5A8HQ absorption spectra as shown in Fig. 8B. 5A8HQ markedly diminished absorption band of the polypeptide backbone as a concentration dependent manner and produced a peak shift to the red from 222 to 225 nm at 50 µM 5A8HQ (Fig. 8B,C). Alteration of the aromatic absorption band was not clearly observed on normal absorption spectra, but it was notably red-shifted (from 278 to 280 nm) on the second-order derivative form. These findings suggested that 5A8HQ interacts with BSA via complex formation, and perturbs amide bonds as well as aromatic amino acids of the BSA to more polarity^53^. This finding was consistent with the synchronous fluorescence and molecular modeling analyses. In addition, the prominent band at 254 nm was observed on the corrected BSA‒5A8HQ spectra, which might belong to photon absorptivity of the bound 5A8HQ. By sitting into the protein cavity, 5A8HQ might be stabilized and shielded from other quenchers, resulting in more energy absorption at its characteristic band.

Structural alteration of the BSA when interacting with 5A8HQ was further elucidated by a CD spectroscopy, which is a useful technique for analyzing the secondary structure of macromolecules. BSA structure is dominantly composed of α-helix (~ 67%), represented by two prominent negative bands at 208 (π–π* transition) and 222 (n–π* transition) nm, and a positive band at 192 nm (π–π* transition)^54^. The characteristic ellipticity of BSA was slightly altered by 5A8HQ (Fig. 8D), where α-helix content of BSA was reduced from 66.17% to 64.96% and 63.65% upon exposure to 5A8HQ at 5 and 50 µM, respectively. This finding is in well agreement with other observations, especially thermodynamic parameters, molecular docking, and molecular dynamics simulations, which implied that BSA tends to transform its native conformation for ground-state complexation with 5A8HQ. Hydrogen bonding and electrostatic networks of the complex might reallocate hydrogen bonding of α-helix structure as indicated by a slight decrease in CD spectral bands. Molecular dynamics simulation later found that some parts of subdomain IIB nearby the 5A8HQ binding site became less fluctuation or more structural compact.

### Exploring the binding site of 5A8HQ on BSA through site probe competitive analysis

Serum albumin can bind and transport various molecules since its structure assembles many binding pockets. Site I and site II are well-defined binding pockets located in hydrophobic regions of subdomains IIA and IIIA, respectively, which primarily bind aromatic and heterocyclic compounds. Site III is found on the hydrophilic region of subdomain IB to serve as a binding pocket for numerous compounds e.g., bilirubin, hemin, and sulfonamide derivatives. In addition, seven binding sites for fatty acids are discovered on subdomains IB, IIIA, and IIIB, and at the interface between subdomains^31^. Herein, site-probe displacement study was employed to determine the preferred binding site on BSA of 5A8HQ using warfarin, ibuprofen, and digitoxin as competitive probes for sites I, II, and III, respectively^55^. Binding constant of 5A8HQ to BSA was determined during equilibration with each site marker (Table 4). Site markers caused twice increase of the binding constants, while still retained the Hill coefficient constant at 1, indicating that 5A8HQ does not directly bind to BSA at the sites I, II, and III, but it may sit nearby those binding sites and slightly be affected with the binding of site markers.

**Table 4:** Binding constant (${K}_{A}$) and number of binding site ($n$) of 5A8HQ to BSA (4 µM) in the presence and the absence of site markers (4 µM) at 310 K. r^2^ is the squared correlation coefficient.

Site marker	${K}_{A} \times$10^4^ (M^−1^)	$n$	r^2^
Without	1.195 ± 0.001	1 ± 0	0.9910
Warfarin	2.124 ± 0.001	1 ± 0	0.9587
Ibuprofen	2.090 ± 0.002	1 ± 0	0.9483
Digitoxin	2.103 ± 0.002	1 ± 0	0.9410

### Molecular docking of 5A8HQ on BSA structure

**Figure 9:**
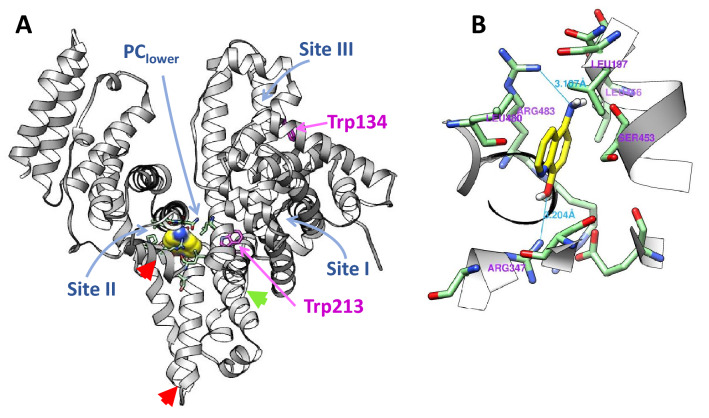
(**A**) Ribbon representation of BSA structure and superimposition of 5A8HQ (yellow surface contour) at the most favorable binding site. Major distortions of structural flexibility are marked by arrowheads. Red arrowheads indicate the decreased fluctuation regions, while green arrowhead refers the increased fluctuation region of BSA upon interaction with 5A8HQ. (**B**) Interaction network among 5A8HQ and amino acid residues of BSA within 5 Å radius. Hydrogen bonds and estimated distances are shown in cyan color.

Chain A of the high-resolution BSA structure was docked with 5A8HQ without water and heteroatom interferences using an automatic AutoDock workflow available at DockingServer^56^. The lowest binding energy (− 5.53 kcal/mol) was obtained, when 5A8HQ was posed on BSA at the lower area of a cleft between subdomains IIA and IIIA (PC_lower_), located adjacent to the Sudlow’s site II and Trp213 (Fig. 9A). This insight was corroborated by the synchronous fluorescence analysis that found an apparent microenvironmental change around Trp. FRET phenomenon also confirmed the proximity among 5A8HQ and Typ residues, which helps reinforce the fluorescence quenching of the static mechanism. The estimated docking binding energy of − 5.53 kcal/mol was equivalent to − 23.14 kJ/mole and very close to the experimentally derived Gibbs free energies at around ‒24 kJ/mole (Table 3). In addition, docking simulation provided comparable results to the CD spectral data and the thermodynamic parameters by Van’t Hoff analysis, indicating that the hydrogen bonding was primarily an interactive force for 5A8HQ‒BSA complex. Amino and hydroxyl moieties of 5A8HQ formed hydrogen bonds with guanidino side chains of Arg483 and Arg347 of BSA (Fig. 9B). Glu499 and Arg348 also exerted their polarity in the interaction. Besides, hydrophobic interaction was observed between quinoline ring of 5A8HQ and hydrophobic side chains of Leu197 and Leu480. These findings were well-concurrent with previous reports, showing that the PC_lower_ pocket of BSA was a preferred binding site for 8HQ derivatives, i.e., 8HQ^57^, cloxyquin^50^, and clioquinol^58^.

### Molecular dynamic simulations of BSA‒5A8HQ complex

**Figure 10:**
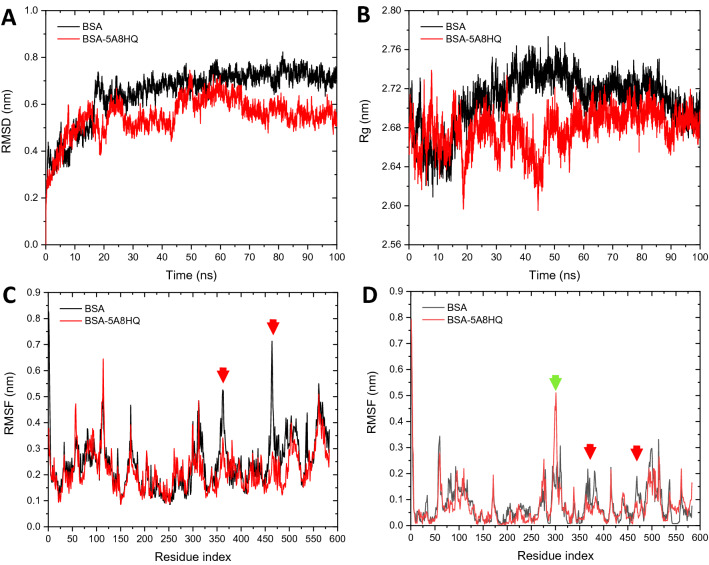
Molecular dynamics simulation results of free BSA and BSA‒5A8HQ complex derived from GROMACS (**A**–**C**) and CABS-flex (**D**) platforms. (**A**) Root mean square deviation (RMSD), (**B**) radius of gyration trajectory (Rg), and (**C**) root mean square fluctuation (RMSF) trajectories from GROMACS. (**D**) Residue fluctuation profile from CABS-flex.

The stability and dynamic property of BSA and its complex with 5A8HQ were simulated on CABS-flex^59^ and GROMACS^60^ platforms under physiological conditions. The structural stabilities of free and 5A8HQ bound BSAs could be realized on the plots of root mean square derivation (RMSD) over time, which reflect the relative conformational changes of each simulation timeframe in comparison with initial posture. GROMACS simulation trajectories within 100 ns revealed that the structural movements of BSA and its 5A8HQ bound form were stable after 50 ns of simulation (Fig. 10A). However, the BSA‒5A8HQ was seemingly more stable than the free BSA as indicated by its smaller average RMSD value (0.59 and 0.72 nm of average RMSD within 50 and 100 ns timespan for 5A8HQ bound and free forms, respectively). Moreover, the stable complexation of BSA with 5A8HQ was also observed on the radius of gyration (Rg) time course (Fig. 10B). The binding of 5A8HQ slightly caused a decrease in Rg values as compared to the free BSA, implying that the secondary structure of BSA became more compact when complexing with 5A8HQ, especially after 20 ns of simulation. The average Rg values during 50 and 100 ns of simulation were 2.68 and 2.72 nm for BSA‒5A8HQ and free BSA, respectively. Additionally, the fluctuation profile of amino acid residues is useful to reveal conformational flexibility and stability at the subdomain level. GROMOS forcefield obviously demonstrated a decrease in the root mean square fluctuation (RMSF) at residues 349–372 and 451–474 of BSA as a result of 5A8HQ binding (Fig. 10C; indicated with red arrowheads). The average RMSF of entire BSA‒5A8HQ structure (0.24 nm) was also slightly smaller than that of free BSA (0.21 nm). Furthermore, CABS-flex package was also applied to study protein residue fluctuation because its simulation outcomes have been proven to be comparable with NMR-derived information and could be used in complement with the atomic molecular dynamic (MD) simulations^61^. CABS-flex simulation provided equivalent residue fluctuation profiles with GROMACS. Once complexation with 5A8HQ, the BSA structure was relatively more rigid as compared to the free form. This observation was indicated by the reduction of an average residue fluctuation about 8.2%. Amino acids of subdomain IIB at the positions 364–371 and 469–476 clearly turned out to be less fluctuation in the presence of 5A8HQ (indicated by the red arrowheads in Figs. 9A,10D). Both GROMACS and CABS-flex simulations suggested that a reduction of residue fluctuation mainly appeared on subdomain IIB nearby the predicted binding site. Therefore, the PC_lower_ region on BSA should be the favorable binding pocket of 5A8HQ. The resulting interaction network also provoked rigidity of the subdomain IIB and maintains stability of the BSA‒5A8HQ complex.

## Conclusion

5A8HQ, an amino derivative of 8-hydroxyquinoline, is a promising anticancer candidate that can overcome bortezomib-resistant cancer cells. To gain insight into pharmacokinetic and pharmacodynamic behaviors of 5A8HQ, physicochemical parameters of 5A8HQ and its interaction with serum protein have been explored using BSA as a serum protein representative. 5A8HQ possessed drug-likeness properties with high water solubility and intestinal absorptivity. Fluorescence quenching studies indicated that 5A8HQ was associated with BSA through the ground-state complex formation with a moderate binding constant at 10^4^ M^−1^, which was comparable to other 8HQ derivatives, such as 2‑amino‑8‑hydroxyquinoline^62^ and 5-chloro-8-hydroxyquinoline^50^, but not clioquinol (5-chloro-7-iodo-8-hydroxyquinoline; ${K}_{A}$= 10^8^ M^−1^)^58^. However, in vitro obtained binding constant is a preliminary information, which may need further exploration in vivo because some endogenous factors can exert synergistically or antagonistically effect on the binding affinity^63^. Molecular docking and molecular dynamics simulations were concurrently suggested that a cleft between subdomains IIA and IIIA nearby the Sudlow’s site II on BSA would be a favorable binding site of 5A8HQ to generate a stable 5A8HQ‒BSA complex. The experimental point of view indicated that 5A8HQ‒BSA complexation was thermodynamically favored process driven by enthalpy because of the hydrogen bonding and electrostatic interactions. Its moderate binding constant was also consistent with the predicted free fraction (0.484) in serum, suggesting that 5A8HQ seems to display good bioavailability in plasma to reach target sites of action, and exerts pharmacological activities.

## Materials and methods

### Materials and reagents

Bovine serum albumin (BSA; 98% purity with essentially fatty acid free), 5-amino-8-hydroxyquinoline (5A8HQ), warfarin, ibuprofen, and digitoxin were obtained from Sigma-Aldrich Co. LLC. (St. Louise, MO, USA). BSA was prepared in Tris–HCl buffer (10 mM Tris–HCl and 150 mM NaCl at pH 7.4) and determined concentration by photometry at 280 nm with a molar extinction coefficient of 43,824 M^−1^ cm^−1^. 5A8HQ and site markers were dissolved in methanol and used without further purification.

### Fluorescence quenching measurements

BSA (4 µM) was equilibrated with various concentrations of 5A8HQ (0, 5, 10, 30, 50, 70, 90, 110, 130, 150, and 170 µM) for 10 min at the different temperatures (290, 300, and 310 K). Later, fluorescence spectra in a range of 285–450 nm were measured with 280 nm excitation using a temperature-controlled QuantaMaster™ 40 spectrofluorometer (Photon Technology International, Inc., Ontario, Canada). Site marker displacement analyses were achieved as the abovementioned, but an equimolar concentration of 5A8HQ was added to BSA prior challenging with various 5A8HQ concentrations at physiological temperature (310 K). Inner filter effect (IFE) becaused by the excitation and emission beam attenuations by sample might produce nonlinearity artifact on the measured fluorescent signal, so IFE was routinely eradicated by the following equation^55^.

9 $${F}_{cor}={F}_{obs}{\times e}^{\frac{({A}^{ex}-{A}^{em})}{2}}$$

where ${F}_{obs}$ and ${F}_{cor}$ are the measured and corrected fluorescence intensities, respectively. ${A}^{ex}$ and ${A}^{em}$ are the absorbance values at excitation and emission wavelengths being measured. The IFE corrected fluorescence signals were served for estimations of all pertinent parameters, such as quenching constant, binding constant, and thermodynamics parameters, if not otherwise stated.

### UV–Vis absorption measurements

A UV-1800 spectrophotometer (Shimadzu Corp., Tokyo, Japan) was used to measure absorption spectra of BSA (4 µM) in equilibration with 5A8HQ (10, 30, and 50 µM) at 310 K using 1 cm path length cuvette at 1 nm slit width.

### Circular dichroism (CD) spectroscopy

CD measurement was performed to determine a secondary conformational alteration of BSA under 5A8HQ exposure using a Jasco J-815 spectropolarimeter (JASCO International Co. Ltd., Tokyo, Japan). BSA (4 µM) was incubated with 5A8HQ (5 and 50 µM) in a 1 mm path length cuvette at 310 K under constantly flushed with nitrogen. The spectra were recorded in a range of 200–260 nm at an interval of 1 nm with a 60 nm/min scanning rate.

### Physicochemical and ADME predictions

SwissADME (http://www.swissadme.ch)^35^ and pkCSM (http://biosig.unimelb.edu.au/pkcsm)^36^ servers were employed for calculation of physicochemical and pharmacokinetic parameters (ADME; absorption, distribution, metabolism, and excretion) of 5A8HQ. Chemical structure of 5A8HQ in SMILE format was submitted to SwissADME and pkCSM for calculations under default setting.

### Molecular docking analyses

BSA crystal structure (2.47 Å) was retrieved from the Protein Databank (PDB; https://www.rcsb.org/) with an accession number of 4A5S. Only chain A of the obtained BSA structure was selected and processed to remove water molecules and heteroatoms for computational studies. 5A8HQ structure was obtained from PubChem database (https://pubchem.ncbi.nlm.nih.gov/) with a CID416002 accession number and appended with Gasteiger charges and hydrogen atoms. The molecular dockings were executed with 0.375 Å spacing in a grid map of 90 × 90 × 90 Å at the x, y, and z coordination center of 8.52, − 21.57, and 106.69, respectively. To obtain all possible conformations, 100 conformations per ligand were generated using the Lamarckian Genetic Algorithm. The calculations were based on an AutoDock tool available as a web service at DockingServer (https://www.dockingserver.com/web)^64^. After docking calculations, UCSF chimera 1.15 (https://www.cgl.ucsf.edu/chimera/) was employed for visualization and analysis of the resulting docking poses.

### Molecular dynamics simulations

Effect of 5A8HQ on flexibility of BSA structure was studied using the CABS-flex 2.0, a webserver for fast simulations of protein structure flexibility based on a coarse-grained protein modeling method (CABS), which is available at http://biocomp.chem.uw.edu.pl/CABSflex2^59^. Structural files in PDB format of BSA and BSA‒5A8HQ were submitted to CABS-flex server under default setting of restraint parameters. Also, molecular dynamics (MD) simulation on BSA‒5A8HQ complex was also carried out using the GROMACS package provided by a WebGRO (https://simlab.uams.edu/)^60^. Briefly, the best docking pose of BSA‒5A8HQ complex was prepared in PDB format and the essential topology files of 5A8HQ ligand were generated using a ProDRG2 server (http://davapc1.bioch.dundee.ac.uk/cgi-bin/prodrg)^65^. Afterward, structural and topology files were co-submitted to WebGRO. MD simulations for both free BSA and BSA‒5A8HQ complex were accomplished with GROMOS96 43a1 forcefield under 0.15 M NaCl solvated SPC water model within a triclinic box. After energy minimization and NVT/NPT equilibrations, MD simulations were executed with the leap-frog integrator for 100 ns under 1.0 bar at 310 K.
